# Understanding Symptom Self-Monitoring Needs Among Postpartum Black Patients: Qualitative Interview Study

**DOI:** 10.2196/47484

**Published:** 2024-04-26

**Authors:** Natalie Benda, Sydney Woode, Stephanie Niño de Rivera, Robin B Kalish, Laura E Riley, Alison Hermann, Ruth Masterson Creber, Eric Costa Pimentel, Jessica S Ancker

**Affiliations:** 1 School of Nursing Columbia University New York, NY United States; 2 Department of Radiology Early Lung and Cardiac Action Program The Mount Sinai Health System New York, NY United States; 3 Department of Obstetrics and Gynecology Weill Cornell Medicine New York, NY United States; 4 Department of Psychiatry Weill Cornell Medicine New York, NY United States; 5 Department of Population Health Sciences Weill Cornell Medicine New York, NY United States; 6 Department of Biomedical Informatics Vanderbilt University Medical Center Nashville, TN United States

**Keywords:** maternal mortality, patient-reported outcomes, patient-reported outcome, health equity, qualitative research, mobile health, mHealth, qualitative, postpartum, postnatal, maternity, maternal, Black, women’s health, ethnic, design need, mortality, death, decision support, information need, informational need, obstetric, obstetrics, mental health, mobile phone

## Abstract

**Background:**

Pregnancy-related death is on the rise in the United States, and there are significant disparities in outcomes for Black patients. Most solutions that address pregnancy-related death are hospital based, which rely on patients recognizing symptoms and seeking care from a health system, an area where many Black patients have reported experiencing bias. There is a need for patient-centered solutions that support and encourage postpartum people to seek care for severe symptoms.

**Objective:**

We aimed to determine the design needs for a mobile health (mHealth) patient-reported outcomes and decision-support system to assist Black patients in assessing when to seek medical care for severe postpartum symptoms. These findings may also support different perinatal populations and minoritized groups in other clinical settings.

**Methods:**

We conducted semistructured interviews with 36 participants—15 (42%) obstetric health professionals, 10 (28%) mental health professionals, and 11 (31%) postpartum Black patients. The interview questions included the following: current practices for symptom monitoring, barriers to and facilitators of effective monitoring, and design requirements for an mHealth system that supports monitoring for severe symptoms. Interviews were audio recorded and transcribed. We analyzed transcripts using directed content analysis and the constant comparative process. We adopted a thematic analysis approach, eliciting themes deductively using conceptual frameworks from health behavior and human information processing, while also allowing new themes to inductively arise from the data. Our team involved multiple coders to promote reliability through a consensus process.

**Results:**

Our findings revealed considerations related to relevant symptom inputs for postpartum support, the drivers that may affect symptom processing, and the design needs for symptom self-monitoring and patient decision-support interventions. First, participants viewed both somatic and psychological symptom inputs as important to capture. Second, self-perception; previous experience; sociocultural, financial, environmental, and health systems–level factors were all perceived to impact how patients processed, made decisions about, and acted upon their symptoms. Third, participants provided recommendations for system design that involved allowing for user control and freedom. They also stressed the importance of careful wording of decision-support messages, such that messages that recommend them to seek care convey urgency but do not provoke anxiety. Alternatively, messages that recommend they may not need care should make the patient feel heard and reassured.

**Conclusions:**

Future solutions for postpartum symptom monitoring should include both somatic and psychological symptoms, which may require combining existing measures to elicit symptoms in a nuanced manner. Solutions should allow for varied, safe interactions to suit individual needs. While mHealth or other apps may not be able to address all the social or financial needs of a person, they may at least provide information, so that patients can easily access other supportive resources.

## Introduction

### Background

This study focused on designing a culturally congruent mobile health (mHealth) app to support postpartum symptom monitoring, as the current practice does not adequately support patients in identifying the warning signs of pregnancy-related death (PRD). First, we describe the public health case for symptom monitoring and decision support for PRD, specifically among US-based, Black patients, a group that faces severe disparities [1,2]. Next, we discuss why the current mechanisms for symptom monitoring and decision support are insufficient. We then outline the existing solutions while also emphasizing the need for new interventions, particularly why those using a combination of mHealth and patient-reported outcomes (PROs) may be appropriate. Finally, we introduce a conceptual model used to accomplish our study objectives.

### PRD and Associated Health Disparities

The pregnancy-related mortality ratio has increased by >200% in the United States in the past 2 decades, and in a recent review of PRDs, experts estimated that 80% of the deaths were preventable [3]. The Centers for Disease Control and Prevention (CDC) defines PRD as “the death of a woman while pregnant or within 1 year of the end of pregnancy from any cause related to or aggravated by the pregnancy” [4,5]. Mental health conditions (22.7%), hemorrhage (13.7%), cardiac and coronary conditions (12.8%), infection (9.2%), thrombotic embolism (8.7%), and cardiomyopathy (8.5%) have been cited as the most common causes for PRD [3]. Although the global maternal mortality rate has declined, the global rates are still high with 287,000 people dying following childbirth in 2020. There are significant disparities in maternal mortality based on a country’s income, with almost 95% of the cases occurring in low- and middle-income countries [6]. Stark disparities in pregnancy-related outcomes in the United States, such as PRD, exist based on race. Specifically, Black or African American (henceforth, referred to as “Black”) perinatal patients experience PRD 3 times more than White perinatal patients [1,2,7-10].

The disparities in maternal health outcomes experienced by Black patients in the United States are based on inequitable access to care, biased treatment, and inadequate communication, driven by systemic racism and all the cascading effects it creates. Black perinatal patients are significantly more likely to be uninsured and significantly less likely to have a usual source of medical care (eg, a primary care clinician) than White patients [7,10]. When Black patients seek care, they face implicit biases that negatively affect care quality and health outcomes [1,7,10-12]. Unsurprisingly, these biases have led to reduced trust in the health care system among Black patients [13-17]. Black patients also receive less patient-centered communication and feel that they have poorer access to communication with their medical team [10,18,19]. Our study aimed to improve the patient centeredness of information and support for Black patients in the postpartum period through a participatory design, an approach by which representative end users are involved throughout the design process [20-23]. While this study focused on Black postpartum patients in the United States, we believe that our findings may provide insights for improving perinatal support for patients from minority groups globally.

### Challenges to Supporting Symptom Recognition and Treatment Seeking Post Partum

Patients encounter several challenges recognizing concerning postpartum symptoms. First, the initial postpartum visit occurs 6 weeks after birth, and 86% of PRD cases occur within the first 6 weeks post partum [24,25]. Second, most strategies for improving postpartum outcomes focus on hospital-based solutions, which rely on people recognizing symptoms and contacting a health professional [7]. Most counseling regarding the warning signs of PRD occurs during the discharge process following delivery, when people are physically exhausted from childbirth and primarily focused on infant care [24]. As such, this is a suboptimal time for patient education about postpartum risk factors. Discharge nurses report spending <10 minutes on the warning signs of postpartum issues, and most nurses could not correctly identify the leading causes of PRD, making it unlikely that their patients could recognize the warning signs [26]. There are many measures for postpartum symptom reporting, but the most common instruments focus narrowly on specific mental health issues, many of which are not specific to postpartum mental health or postpartum health–related quality of life [27]. While these are helpful measures to use in a clinic or hospital setting, they do not provide real-time decision support regarding the full spectrum of severe symptoms that may be indicative of PRD.

### Suitability of Different Solutions for Supporting Symptom Monitoring

mHealth can address the need for tailored, dynamic symptom monitoring and support. The Association of Women’s Health, Obstetric, and Neonatal Nurses and the CDC have developed 1-page summaries to help patients identify the warning signs of PRD, such as the Urgent Maternal Warning Signs (UWS) [28,29]. These tools represent a positive step toward improving symptom management, but these solutions do not provide real-time, tailored support. Telephone-based support staffed by health professionals has been demonstrated to decrease postpartum depression and improve maternal self-efficacy [30-33]. However, 24-hour hotlines can be resource intensive, and people may still experience bias when accessing these services. The goal of this study was to conduct a qualitative needs assessment for the Maternal Outcome Monitoring and Support app, an mHealth system using PROs to provide decision support for postpartum symptom monitoring.

Mobile phones offer a viable, inclusive option for intervention delivery for Black people of childbearing age. In 2020, data from the Pew Research Center indicate that 83% of Black people owned smartphones, which is comparable to smartphone ownership among White people (85%). Smartphone ownership is also higher among people aged <50 years (96%), which encompasses most postpartum patients [34]. However, Black people are twice as likely as White people to be dependent on smartphones for internet access [35]. mHealth-based apps for blood pressure and weight tracking during pregnancy have demonstrated success among diverse groups, providing evidence that mHealth may be an acceptable means for symptom reporting in the target population [36-38].

Symptom education and PRO-based interventions have demonstrated success in improving knowledge, self-efficacy, and outcomes. Use of PROs has improved symptom knowledge, health awareness, communication with health care professionals, and prioritization of symptoms in patients with chronic disease and cancer [39-44]. Multiple studies have also demonstrated that educational interventions regarding expected symptoms in the postpartum period can improve self-efficacy, resourcefulness, breastfeeding practices, and mental health [12,38,45-47]. However, given the issues related to trust and disparities in patient-centered communication, it is critical to understand Black patients’ perspectives about how such a system should be designed and implemented.

### Conceptual Model

**Figure 1:**
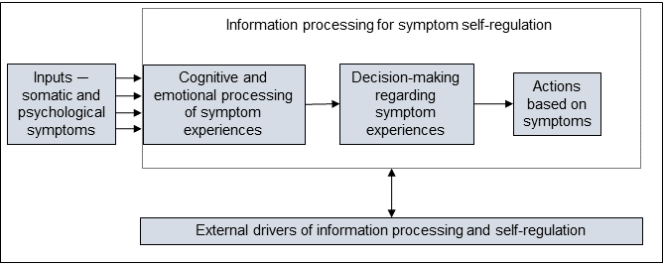
Theoretical model for postpartum symptom processing and self-regulation.

To study the issue of supporting symptom monitoring, we combined 2 theoretical frameworks (Figure 1): the common sense model of self-regulation (health behavior) by Diefenbach and Leventhal [48] and the model of human information processing (human factors engineering) by Wickens [49]. The model by Diefenbach and Leventhal [48] depicts patients as active problem solvers with a mental model of their conditions. Patients process their symptoms, both cognitively and emotionally, and then evaluate whether action is needed [48]. The patient’s mental model of their condition, personal experiences, and sociocultural factors impact processing, evaluation, and action. In the information processing model by Wickens [49], action occurs in 2 steps—selection and execution [48]. Environmental or organizational factors also affect patients’ selection of actions and whether they can execute an action. For example, a patient may suspect that they should visit the emergency room but may not go because they do not have insurance, transportation, or childcare. Our qualitative inquiry investigated how to better support symptom processing and appropriate response selection, while also uncovering the barriers to action that may need to be mitigated.

### Objectives

The goal of this study was to identify the design and implementation needs of an mHealth-based symptom self-monitoring and decision-support system to support Black patients in determining when to seek care from a health professional for signs of PRD in the postpartum period. This tool will support both somatic and psychological symptoms given their complex, critical, and connected presentation. We used the described conceptual model in qualitative inquiry and pragmatic intervention design to provide contributions regarding the following: (1) relevant symptom inputs for postpartum support, (2) drivers that may affect symptom processing, and (3) how the previous 2 aspects highlight the design needs for symptom self-monitoring and patient decision support. To address our study objective, we conducted semistructured interviews with postpartum Black patients, obstetrics health professionals, and mental health professionals.

## Methods

### Setting

The study was conducted in 3 tertiary care hospitals and affiliated clinics within the same health system in New York City. The 3 hospitals, taken together, are involved in the delivery of >14,000 babies annually. All participants were either patients who received obstetric care in the included sites or health professionals affiliated with the sites.

Eligible patients were identified by the institutions’ research informatics team using electronic health record data. First, the patients’ providers consented to their patients being contacted, and patients’ charts were reviewed by the primary obstetrician or designate to ensure that the patient was eligible for the study and that they had a delivery experience that would allow them to participate in the interview without undue stress. Next, the patients were sent an invitation to participate via the email address listed in their record. We also posted fliers in 2 high-risk, outpatient obstetric clinics.

Obstetric and mental health professionals were eligible if they were affiliated with one of the institutions in the obstetrics or mental health department. Brief presentations were given at relevant faculty meetings, and participants were contacted individually via email or through departmental listserves.

Interested participants from all groups used a link to schedule a time to speak with a researcher.

### Ethical Considerations

The study was approved by the affiliated medical schools’ institutional review board (protocol number 20-08022582). All participants provided written informed consent. Study data were coded (ie, all identifying information was removed) to protect participant privacy. Each participant was compensated US $50 for their time via a physical or electronic gift card.

### Study Design and Sample

The study used semistructured interviews with 3 key stakeholder groups: recent postpartum Black patients, obstetric health professionals, and mental health professionals. Eligible patients were within 12 months post partum of a live birth, self-identified their race as Black or African American, and had at least 1 somatic or psychological high-risk feature associated with their pregnancy. High-risk features included attendance at a high-risk clinic for prenatal or postnatal care, inpatient hospitalization within 12 months post partum, a prescription of an antidepressant or benzodiazepine within 12 months of the pregnancy, or a new diagnosis of depression or anxiety within 12 months of the pregnancy. High-risk clinics treated various conditions, but the most common conditions were gestational hypertension and gestational diabetes.

We adopted an interpretivist qualitative research paradigm to study patient and health professionals’ perspectives of how symptom recognition and care seeking may be better supported [50]. Our methodological orientation involved directed content analysis, adopting an abductive reasoning approach. First, we used the previously specified conceptual model to construct questions and thematically categorize responses [48]. Then, we allowed unique subthemes to inductively emerge from the data collected [51].

### Interview Guide Development

Interview guides were iteratively developed by our team of researchers with expertise in obstetrics, perinatal mental health, nursing, consumer informatics, inclusive design, and qualitative methods. The guide for each stakeholder group was reviewed and piloted before enrollment of the first participant. Interview guides were tailored for patients or health professionals but followed a similar structure, based on our conceptual model (Figure 1), such that participants were first asked about barriers to and facilitators of processing symptoms cognitively and emotionally (eg, Do they notice the symptom or realize its severity?), making decisions about symptoms they are experiencing (ie, When to seek help from a health professional?), and taking action on problematic symptoms. Probing questions encouraged participants to elaborate on experiential, educational, sociocultural, organizational, environmental, or health systems–level drivers of patients’ symptom management. Then, participants were asked a series of questions related to their thoughts regarding the design of the mHealth system, including how to best report symptoms, the wording of system decision support, the desired level of involvement of the obstetrics health professionals, the means for facilitating outreach to a health professional, additional information resources, and preferences for sharing information included in the system with a trusted friend or family members. During this process, obstetrics and mental health professionals were also shown a handout that outlined the draft of the symptom management algorithm for the system being developed (CDC’s UWS) and asked if they would make any changes, additions, or deletions [29]. Full interview guides are included in Multimedia Appendix 1.

### Data Collection

All interviewees provided consent electronically before the interview. A PhD-trained qualitative research expert (NB) completing a postdoctoral study in health informatics and population health conducted all the interviews via Zoom (Zoom Video Communications) or telephone. Participants had the option to request an in-person interview, but none of them chose this option. Interviews lasted 30 to 60 minutes and were audio recorded. We explicitly described the study objectives to each participant before the interview. Following the interview, participants completed a demographics survey electronically. All electronic survey information was collected using REDCap (Research Electronic Data Capture; Vanderbilt University).

### Data Preparation and Analysis

Audio recordings were converted into transcripts using an electronic software (NVivo Transcription; QSR International) and manually checked for accuracy by a study team member who did not conduct the initial interviews. We completed all data analyses using NVivo (versions 12 and 13), but we manually analyzed the data and did not use computer-aided techniques (eg, computerized emotion detection or autocoding).

Data were analyzed using thematic analysis and the constant comparative process [51-53]. Specifically, each analyst open coded the transcripts, by coding segments that pertained to the research questions, as opposed to coding all words and phrases. We used thematic analysis to detect the common and divergent needs for postpartum symptom monitoring. We chose this method over other approaches such as grounded theory or sentiment analysis because our needs were pragmatic to solution design, and we were not attempting to establish theory, describe phenomena, or represent collective feeling about a topic.

The first deductive analysis was conducted using an initial theoretical model derived from the common sense model by Diefenbach and Leventhal [48] and the model of human information processing by Wickens [49] (Figure 1). To promote reliability, 2 coders in addition to the interviewer were involved in the analysis, and each transcript was first analyzed independently by at least 2 people (NB, SW, or SNdR), followed by meetings to resolve discrepancies based on consensus coding. The analysis team created initial codes based on the conceptual model and added new items to the codebook inductively (ie, post hoc instead of a priori, as they arose in the data). The team used NVivo to maintain a working codebook of themes, definitions, and relevant quotes derived from the data. The codebook was periodically presented to coinvestigators with expertise in obstetrics and perinatal psychiatry to improve external validity [51,52]. The sufficiency of sample size was assessed according to the theoretical saturation of themes encountered, specifically based on the need to add additional subthemes to the codebook [54,55]. After all the transcripts had been coded, at least 2 members of the coding team reviewed the data code by code to ensure that meaning remained consistent throughout the analysis and to derive key emerging themes [51].

## Results

### Participant Characteristics

This study included 36 participants—15 (42%) obstetrics health professionals, 10 (28%) mental health professionals, and 11 (31%) recent postpartum Black patients. Table 1 presents the self-reported demographic information. As shown, 19% (7/36) of the health professionals and 11% (4/36) of the patients had missing data (ie, did not complete the questionnaire). Participants could also selectively choose not to answer questions. “Other” affiliations were possible for health professionals because those who had a secondary affiliation with one of the included sites but primary affiliation with another organization were eligible.

**Table 1:** Summary of the study participants’ self-identified characteristics collected using the survey immediately following their interviews.

Characteristics			Total (N=36), n (%)		Health professionals (n=25), n (%)		Patients (n=11), n (%)
**Health professional role**							
	Advanced practice provider (eg, nurse practitioner or midwife)	4 (11)		4 (16)		0 (0)	
	Attending physician	7 (18)		7 (28)		0 (0)	
	Licensed clinical social worker	2 (5)		2 (8)		0 (0)	
	N/A^a^	13 (34)		0 (0)		11 (100)	
	Nurse	1 (3)		1 (4)		0 (0)	
	Other^b^	4 (11)		4 (16)		0 (0)	
	Missing	5 (14)		7 (28)		0 (0)	
**Affiliation or point of care**							
	Other	1 (3)		1 (4)		0 (0)	
	Site 1	23 (61)		14 (56)		9 (69)	
	Site 2	1 (3)		1 (4)		0 (0)	
	Site 3	2 (5)		2 (8)		0 (0)	
	Missing	9 (25)		7 (28)		2 (18)	
**Gender**							
	Female	26 (68)		16 (64)		10 (77)	
	Male	2 (5)		2 (8)		0 (0)	
	Missing	8 (22)		7 (28)		1 (9)	
**Year of birth**							
	1965-1980	6 (16)		5 (20)		1 (8)	
	1981-1996	22 (58)		13 (52)		9 (69)	
	Missing	8 (22)		7 (28)		1 (9)	
**Race**							
	American Indian or Alaska Native Asian	1 (3)		0 (0)		1 (8)	
	Black or African American	11 (29)		2 (8)		9 (69)	
	Native Hawaiian or other Pacific Islander	1 (3)		1 (4)		0 (0)	
	White	11 (29)		11 (44)		0 (0)	
	Missing	12 (33)		11 (44)		1 (9)	
**Ethnicity**							
	Hispanic or Latino	1 (3)		1 (4)		0 (0)	
	Not Hispanic or Latino	25 (66)		16 (64)		9 (69)	
	Missing	10 (28)		8 (32)		2 (18)	

^a^N/A: not applicable.

^b^Health professionals’ self-reported role of resident psychiatrist, chief resident in psychiatry, psychologist, and patient care director was combined into the *other* category for analysis purposes.

### Structure of Themes

#### Overview

Our initial theoretical model, derived from the common sense model by Diefenbach and Leventhal [48] and the model of human information processing by Wickens [49] (Figure 1), described that patients experience some inputs (psychological and somatic symptoms of PRD). Then, there is a series of drivers that affect how patients cognitively and emotionally process (eg, notice and realize symptom severity), make decisions about, and act on symptoms they are experiencing. The nature of these symptoms, how they are processed, how decisions are made, and how they are acted upon then drive a conversation regarding the design needs for symptom monitoring and decision support for PRD. The emerging themes were organized into the following categories: (1) symptoms of PRD; (2) drivers of processing, decision-making, and action; and (3) design needs for a symptom-reporting and decision-support system. Quotes are labeled with study-specific identifiers: OB denotes obstetric health professional, MHP denotes mental health professional, and PT denotes patient.

#### Inputs: Psychological and Somatic Symptoms of PRD

Concerning and routine symptoms were reported both from a psychological and somatic perspective. Sometimes, the distinction between routine and concerning symptoms was clear. Other times, it was more challenging to differentiate routine versus concerning symptoms particularly because they were related to psychological health. Mental health professionals also noted the challenge that routine symptoms can progress to something more serious over time:

In my mind, like normal becomes abnormal, when there is any kind of functioning [loss] that like withstands two to three weeks.

MHP 04

We really hear a lot about postpartum depression and stuff...A lot of women think...postpartum depression is you just don’t want to. You don’t have it. You go into depression where you can’t take care of your child and you don’t want to hold your child. You don’t feel connected to your child. And I learned...it can be so many different things.

PT 09

A clear distinction was not always present between psychological and somatic symptoms:

If someone...has pain in their chest or shortness of breath, the first thing you want to think about is it sort of like clots and other kind of physiologic reasons for that. Those are also very implicated and sort of obviously [associated with] panic attacks and anxiety. So, I think though those symptoms are also relevant of physical symptoms, [they] are also relevant for mental health.

MHP 05

#### Drivers of Processing, Decision-Making, and Action Based on the Symptoms Experienced

Several drivers were reported to affect symptom processing (ie, whether they noticed the symptom and its severity), patients’ capacity to decide what should be done (ie, make decisions), and whether they were able to act on concerning symptoms (Table 2).

Table 2 presents exemplary quotes for emerging themes under a single driver, but many quotes were coded under multiple drivers in our analysis process. The following passage, for example, highlights how self-perception, sociocultural concerns, and the health system can overlap to present a complex set of factors that may prevent women from receiving the care they need for the symptoms they are experiencing:

A lot of times I think that does get overlooked because people feel like, well, you’re OK, you’re fine. But what research shows us is that especially for Black women, it really doesn’t matter how much money you make or your income level, like our postpartum and perinatal health outcomes are the same across the board, which is really detrimental. So, yeah, I think they get overlooked because of that. I think they get overlooked or we get overlooked in the health care system. But I also think we get overlooked by our family and friends because we’re the strong ones. So, if anybody can deal with this, it’s you.

MHP 10

**Table 2:** Descriptions of the drivers of symptom processing, decision-making regarding symptoms, and taking action based on symptoms patients’ experience in the postpartum period.

Drivers	Description of emerging themes	Example quotes
Self-perception	Health professionals and patients described the challenges in recognizing symptoms based on a lack of knowledge regarding what is normal during the perinatal period and the continuous focus on their newborn, not allowing time for self-reflection.	“But I would say...one of the hard things I think for women is, especially for first time mothers, they don’t know what it’s supposed to feel like postpartum. They have nothing to compare it to...And I find that that’s the biggest barrier for women seeking out care, is that they just don’t know what’s normal or not normal.” [MHP^a^ 02] “I was very much attached to her [patient’s child], but you just felt like you felt down, and you felt like, am I doing enough? So, I never really felt like I was doing enough. Um, and you’re exhausted, right? So, when you’re exhausted can’t process too much.” [PT^b^ 13]
Previous experience	Patients’ previous experiences could either help them know what to look for or could prevent them from seeking help, if their previous experience was negative.	“It’s kind of hard to say because I already know what to look out for because I had postpartum with my first kid. So, I kind of already know I didn’t really need much education in that area.” [PT 05]
Sociocultural factors	Social factors could be supportive or prohibitive based on how the patients’ support system viewed seeking help, particularly for mental health. Participants also explained how stigma plays a strong role in processing symptoms and seeking help for mental health–related issues. Participants articulated that social factors may be influenced by cultural norms based on age, race, country of origin, or previous experiences with mental health. Lack of social support (ie, not having someone to help with childcare while seeking medical attention) was also described as a barrier to acting upon concerning symptoms.	“On one hand...the patient might not have full insight into how they’re doing and what’s going on for them, and the partner might have a lot of insight...It could be very positive to have that information. At the same time, I have also seen partners who then either exaggerate or pathologize them or don’t understand some of the changes...but are just part of becoming more preoccupied in that phase with the infant and infants care and that status parenthood. And so that can be a very...difficult and alienating experience for the mother.” [MHP 06] “I live with my son’s father...He was there, so he was the one that told me to go. And I told my mom, she was like, I should go in. And that’s when I called the doctors because it was a little concerning.” [PT 01] “They feel like they should be able to do it. They feel like this is, you know, other women do this and they don’t have any issues. So, why am I having difficulty with this?...They feel like they’re weak, they’re incapable, or incompetent. And yeah, that’s definitely a barrier.” [MHP 02] “I’m thinking of one patient who, like, she was really like every time was so close, had her see a therapist and had her see me...The location background, really, like grew up in an environment where like, psychiatry is just not something that you do and you should tough it out, which was so impaired, like this is like years and years and like, it just got to be more severe.” [MHP 01] “I think there’s just so much stigma and shame about having difficulties in that initial period of time.” [MHP 03] “And I think that’s like the most difficult thing for them to deal with, especially if, like the father of the child is not involved or like there’s no family in the area. And I don’t honestly see it very often. But when I when there is an issue with, like someone being admitted or someone coming in, it’s usually because there’s another child.” [OB^c^ 03]
Financial and insurance-related factors	Financial and insurance-related factors were described as potential barriers to seeking care. Cost-related issues were particularly prevalent during discussions about mental health.	“So, I think there’s a fractured system. I think we’re kind of isolated and working in our silos to a large extent, and then if we really talk about mental health...the big ugly secret is we don’t take insurance. Most people you go on Psychology Today and you look for perinatal, these people are not taking insurance and they’re certainly not taking Medicare or Medicaid.” [MHP 08] “So, the first one is insurance and the cost of care, which is a big one, and a lot of people will choose not to go to therapy because it’s not affordable.” [MHP 07]
Built environment	Issues related to the patient’s environment such as how far away they lived, traffic, and parking were described as barriers to seeking and receiving care.	“Like traffic is like never ideal, you know, during certain times of the day. Parking in the area is not ideal...So, like, I can only imagine that, like especially like immediately postpartum with like a crying baby at home, like I have to drive into the city.” [OB 03] “Some patients use public transportation if they live in the boroughs. And so, they don’t have access to a vehicle that they can easily get back to the hospital.” [OB 04]
Health system–related factors	There were various levels of health system factors that could impact how patients processed symptoms, made decisions, and took action. Patients described individual positive experiences of how processes and their care team prepared them for the types of symptoms they should look out for. However, health professionals described anecdotes of how patients’ previous experience of bias and systemic racism prevented women from seeking care.	“When I was being discharged the first time, they were very clear about what numbers should be alarming or would be cause for concern. And so, I had my discharge papers, they were handy. But by that point, I think it was just pretty much ingrained what the numbers I should be looking for were. But...the discharge papers were very clear.” [PT 02] “Yeah, patients can tell when they’re under a microscope. And especially Black women are scared when they come into the hospital. They know that Black women are more at risk of dying in the hospital...and that their needs will not be heard, that their expressions for pain or need will be ignored...or they generally just mistrust all of us.” [MHP 07] “There is one attending who is very involved in how patients are feeling and their concerns and everything and her patients happen. Her patient was Black and she just had a lot of anxiety. She just kept saying, If I go to the hospital, am I going to die?...You’re supposed to go to the hospital to get the help and the care you need, but if you go to the hospital, are you going to die? You know, that’s kind of really sad that a patient would feel that way, you know, especially at such an exciting time in her life...So, with that stigma out there, I wouldn’t want something like that to have patients avoid getting care because they think they’re better off just not going to the hospital and not going to be hurt.” [OB 07]

^a^MHP: mental health professional.

^b^PT: patient.

^c^OB: obstetric health professional.

#### Design Needs for a Symptom-Reporting and Decision-Support System

Obstetric health professionals, mental health professionals, and patients discussed multiple needs for improved PRD symptom reporting and decision support. The key design requirements are embedded and italicized in the following text.

Participants generally agreed that although the proposed system focuses on postpartum symptoms, *it would be advantageous to introduce the system during pregnancy, particularly in the third trimester*:

You have to reach women before they give birth. They might look, they might not look, they might look at it and be concerned. But then they might forget about it and not have time to call. Those first six weeks are really chaotic.

MHP 06

I think in the third trimester would be great because often we don’t really have anything to talk about in the office. It’s very quick visits like blood pressure and you’re still pregnant and we’re just waiting. And so, I think and they start to have a lot of questions about like, well, when I get home and how’s this going to go? So, I think that time is a good time. We’re all kind of just waiting for labor to happen or full term to get there, and this kind of gives them something to feel like they can prepare for.

OB 08

Patients were open to reminders regarding entering symptoms they were experiencing, and participants described a desire for just-in-time symptom reporting and decision support, so that they could get quick feedback as they were experiencing the symptoms:

When people get home so much in their life has changed. And it’s probably a very hectic time. So maybe I think that’s a great idea reaching out again, either a few days or a week later to make sure they’re really able to use it and engage with it to the extent that’s helpful to them.

OB 02

I think it would be a good idea to have like a system where you can report whenever you want.

PT 03

I think for me, I would say in the moment. But then also having something at the end of every week to just, you know, to check in with yourself. I think that would be good as well.

PT 09

In addition to considerations about how symptoms would be recorded, participants stressed the *importance of the wording of the decision-support messages that patients receive*. For messages that inform the patient that their symptom did not seem to require immediate medical attention, *it was important to ensure that the patient still felt heard and that they did not leave the interaction feeling stuck* with nothing to do regarding a symptom that was concerning to them:

Reframe the message. You know...we apologize that you were experiencing this. We just want to reassure you that this is normal.

PT 01

[You] don’t want to make anyone feel like their feelings aren’t valid because that’s a horrible thing, especially in health care, especially if a person is convinced that something is wrong with them and you’re telling them that it’s normal and is perfectly fine. So, in that situation, I would just, depending on what the issue is, I would also share information of what to look out for.

PT 05

The first thing is that it’s normal, but also something that you want to be able to do for comfort. For me, I don’t have to do too much, especially if I’m having anxiety, like if I get a text back that says here are some things you can do in this very moment to handle it. And then also, here are some links or information that you can also look up.

PT 09

In the events where a concerning symptom was reported and it was recommended that the patient should reach out to a health professional, importance of conveying a sense of urgency without scaring the patient:

You don’t want to scare people, but it’s kind of hard to get around that when something is serious, and you don’t want to dumb it down.

PT 01

Participants wanted multiple, easy-to-do methods for connecting with their health professional team, including having the number to call pop up, scheduling a time for someone to call them, and being able to start a live web-based chat:

I like all the options, especially that form or chat you can have like, you know, those online chat where like you really chatting with someone for those who like the type. I’m the type of person I just want to make a phone call, right? So, like for me, [it] will be a call. Maybe say maybe if it’s five, five or ten minutes then that will be great. Like especially, it’s going to make me feel like, OK, there’s someone out there that will care about my health.

PT 06

However, participants noted that they would prefer not to use a symptom-reporting and decision-support tool, but instead reach out directly via phone if they were experiencing issues.

Participants, particularly mental health professionals, described a *need for improved nuance or details regarding the different psychological symptoms patients could experience* that are indicative of severe mental health issues:

Thoughts of hurting yourself or someone else is a good one...I would say I would add difficulty bonding. It would add something about not being able to sleep, even if you could sleep, you know, like or your anxiety that doesn’t go away, that changes your behavior. So, it changes the way that you interact with the baby or kind of do childcare. I guess I would want to say something about. psychotic thoughts, like fear that someone else may be hurting you or...recurrent worries or anxieties that don’t go away.

MHP 02

Patients had differing opinions regarding whether the system should be integrated with other health technologies, particularly the patient portal:

I love the patient portal. I was able to be traveling to reach out to my OB, to reach out to all, you know, the nurses and stuff like that and just experience things that I needed.

PT 09

I feel like...it’s an integral part of my medical history. So, even if it may seem somewhat insignificant for whatever reason, I would still want to have access.

PT 09

I didn’t find it [the patient portal] very helpful...

PT 03

On the basis of the feedback from health professionals that it may be challenging for postpartum patients to process and recognize certain symptoms, especially those related to mental health, we explored whether patient participants would be open to sharing educational information about symptoms to expect (rather than sharing the actual symptom reports) with trusted friends or family members. Similar to other design considerations, results were mixed, but it seemed helpful to have a *patient-driven option for sharing symptom-related educational information with chosen friends or family members*:

I think that there’s so much going on it would help to have someone with a different perspective equipped with this information.

PT 02

There’s a lot of shame that comes with this. I’m not sure people would actually want other people to know. I can’t speak for the majority, but I didn’t really want people to know because I don’t want the kind of energy that came with people knowing.

PT 05

We also discovered the competing needs of *balancing the patient’s desire for their health professionals to be involved in symptom reporting with the need to avoid significant increases to health professional workload*:

I sort of wonder from the health care provider perspective, how involved is the provider in that in the app? Like, do they get like a PDF of all the information? Is that more work for the provider? How does the provider interpret that data?

MHP 03

I feel like they [the health professional] should be super involved. Especially because I’m not just going off of my experience because, you know, I don’t want to feel like they’re not really like I’m experiencing. And so, it’s scaring me. So, I just want to know that, you know, you’re hands on with everything.

PT 01

Finally, *the participants desired information beyond PRD symptoms to entice them to use the system*. They were supportive of including various types of information, such as breastfeeding support resources, milestones and information regarding their child, other websites and apps with trusted maternal and child health information, further support resources for how they feel mentally, and links to social services (eg, food, housing, or other assistance).

## Discussion

### Principal Findings

In this qualitative study, we interviewed obstetric health professionals, mental health professionals, and Black postpartum patients. Our findings helped to identify the design and implementation needs of an mHealth-based, symptom self-monitoring and decision-support system designed to support Black patients in determining when to seek care from a health professional for signs of PRD in the postpartum period. We encountered important findings related to (1) inputs, including psychological and somatic symptoms; (2) drivers of processing, decision-making, and action based on the symptoms experienced; and (3) design needs for a symptom-reporting and decision-support system. We have discussed how our findings may be helpful to other postpartum populations as well as the implications of our study for patient decision-support in other clinical settings.

First, our findings related to symptom inputs revealed the challenges caused by the overlapping presentation of somatic and psychological symptoms. This provides support for our approach of including psychological and somatic issues in a single app, particularly given that mental health conditions are a leading cause of PRD. A 2021 review found 15 PRO measures for assessing postpartum recovery. The measures typically focused on mental health or health-related quality of life, but few included both psychological and somatic outcomes, and none were targeted for PRD, such as the system [56].

Moreover, related to symptom inputs, we found that current tools for pinpointing severe symptoms, such as the CDC’s UWS did not provide sufficient nuance for concerning psychological symptoms. Symptom-reporting tools for PRD will either need to consider incorporating structured assessments, such as the Edinburgh Postnatal Depression Scale (EPDS) [56], or incorporating additional symptoms. The latter approach may have advantages as the EPDS focuses on depression (while providing subscales for anxiety) and PROs evaluated for use with anxiety disorders have limitations [57]. Furthermore, the EPDS has been validated in in-person laboratory settings but not in community settings or for web-based entry [58]. We must also consider how mistrust in the health system may lead to less truthful answers. Issues expressed around stigma related to mental health indicate that the way in which these symptoms are elicited may require further assessment to promote the normalcy of the symptoms and improve candid reporting. Technology-based approaches for supporting perinatal mental health have been described as uniformly positive but having limited evidence for use [59], suggesting that further exploration is needed in this area, also considering how adding somatic issues may be perceived by patients.

Second, there were several drivers that affected symptom processing, decision-making, and action that cannot typically be solved through a symptom-reporting and decision-support system. Challenges related to self-perception and lack of experience or expectations may be addressed based on the wording for how the symptoms are elicited and by providing concise, easy-to-understand depictions of what should be expected versus what are the causes for concern. However, many of the other issues described related to sociocultural, financial, and environmental factors and the health systems’ systemic racism issues cannot be addressed directly in a simple PRO-based app and decision-support system. Directly addressing these issues will likely require more systematic, multipronged approaches. Therefore, it seems advisable to couple patient decision-support aids with other social support interventions for perinatal health [60,61].

Drivers of processing, decision-making, and action are still important contextual elements to be considered in the design of the system. Another study tailoring an mHealth app for Latina patients to support health during pregnancy also found it important to address issues related to financial barriers, social support, health care accessibility, and cultural differences [62]. Our best attempt to address these issues may be to promote information transparency and inclusive design. For example, there may be a “frequently asked questions” section of an app, where patients can explore things such as supportive resources for childcare while they seek medical attention or information they may show their friends or family members regarding postpartum symptoms of concern. The system may also use common human-computer interaction principles, such as information filtering [63] and organizing the suggested resources (eg, for mental health care) based on whether they accept the patient’s insurance. The built environment can also be changed through the system, but it may offer mechanisms for remote monitoring, such as telemedicine-based support or linking the system to a blood pressure cuff, when clinically appropriate [64,65]. As noted, the system obviously cannot address issues related to systematic racism directly [66]. Instead, we used a participatory design approach, with the hope that the nature of the information presented may be more patient centered, acceptable, and better aligned with the beliefs and values of Black patients [67]. Issues related to systematic racism have commonly been described in the US health care system, but structural inequities also exist on a global scale. Future studies should investigate how our findings regarding design needs may extend to other minoritized perinatal patient groups.

A systematic review of patient decision aids for socially disadvantaged populations across clinical settings found that such tools can improve knowledge, enhance patient-clinician communication, and reduce decisional conflict [68]. However, descriptions of patient decision aids focus on the type of tool (eg, paper vs digital), how it was delivered, when it was delivered, and by whom, as opposed to describing the content the aid provides. Therefore, it is challenging to determine how other decision-support tools have addressed information regarding environmental, financial, or health system–level factors that may affect care seeking based on the decision aid. Some tools seem to address sociocultural needs by tailoring to the target population, but the aforementioned systematic review did not find differential effects on outcomes when tools were tailored versus not tailored [16]. Future studies on patient decision aids may benefit from including non-symptom related information. Providing appropriate informational support may involve a deeper study of the systemic needs that patients may have, even if these needs may not directly be addressed by the decision aid.

Third, descriptions of the design needs for PRD symptom monitoring revealed that there is likely not a one-size-fits-all solution related to reminders, involvement of health professionals, and how the tool is incorporated with other systems (eg, the patient portal). “User control and freedom” and “flexibility of use” are two of the key items in commonly used heuristics for user interface design [69]; therefore, it is important to include options for customization and varied but safe pathways for interaction with the proposed system. For example, some participants described that they may not be likely to access the symptom-reporting system through the patient portal. Although there may be safety and convenience-related reasons for having the system as part of the patients’ medical record, if the patient chooses, the system could, on the front end, appear more like a stand-alone app than something that must be accessed through the patient portal. Patients also had varying opinions related to how they may want to reach out to a health professional if a problematic symptom was reported. These preferences may differ from instance to instance; therefore, it is helpful to ensure that patients have a choice regarding how to reach out, but system designers must also create workflows with feedback loop, so that patients who are reporting problematic symptoms are not missed (ie, if patients do not reach out themselves, they never receive attention). Patient-level customizations and options for interaction also respects patients as individuals and may promote patient-centered interactions.

Furthermore, related to design needs, participants indicated that the wording of the decision-support messages was critical. Specifically, for reports that did not include currently urgent symptoms, it was important that the message still conveyed support and validation, clarified that the patient could still reach out for help, and provided additional means for managing their symptoms, so the patient did not feel frustrated by their report [70]. Regarding messages that recommended patients to reach out to their health professional team, it was crucial to note what the symptom meant (eg, what kind of disease it could indicate), encourage the patient to reach out without increasing anxiety, and provide different avenues for easy outreach. Going forward, we plan to incorporate the aforementioned elements into the messages built into the system. We will then complete additional acceptance and comprehension testing with a larger sample of postpartum patients. These findings also indicate that care must be taken in translating such tools, and the translated materials should be reviewed with the target end user groups before implementation. This may mitigate unintended consequences or inadvertent inclusion of language that does not support the needs of minoritized groups.

### Strengths and Limitations

Our study highlighted the limitations and areas that would benefit from further exploration. First, our study involved recruitment sites that were within a single health system in New York City. Second, while we achieved thematic saturation of qualitative themes (a means for determining sample sufficiency in qualitative studies) [54,55], our conclusions are based on a sample of 36 participants from 3 stakeholder groups. Third, given the documented disparities, we deliberately focused on the needs of Black postpartum patients, but this may not represent the needs of the postpartum patients of other races. Furthermore, our sample should not be viewed as encompassing the opinions of all Black postpartum patients. Our findings revealed the need for individual customization and varied interaction patterns on a case-by-case basis. Fourth, all interviews were conducted remotely (via Zoom or telephone), which can have effects on the interaction. On the one hand, it may be harder to connect with the interviewee, and on the other hand, people may feel more anonymous and comfortable with sharing information. Finally, although we attempted to promote external validity through the review of the coding scheme by a subject matter expert, we did not have the opportunity to perform triangulation of the findings by returning the results to participants. To address these limitations, it would be beneficial to survey a larger group of postpartum patients, powered to assess the differences based on race and ethnicity. This would allow us to come to a stronger consensus regarding design choices, assess whether there are differences in design needs or preferences, and gain feedback from patients in areas outside New York City. Future studies may also explore how other underserved groups, such as those with limited English proficiency, may benefit from tailored symptom self-monitoring and decision support.

### Conclusions

In this qualitative study regarding postpartum symptom monitoring and decision support, we found that the current structured reporting measures do not include the combination of somatic and psychological symptoms that may be indicative of severe outcomes in the postpartum period. While not explicitly related to symptom reporting and decision support, patient decision aids, particularly those focusing on minoritized groups, should consider how the aids may be coupled with other structural support interventions or, at least, information about how other resources may be accessed. As stated in the commonly accepted design heuristics, we also found that user control and freedom unsurprisingly remain important for a patient decision-support aid for Black postpartum patients. Finally, decision aid–related phrases must take care to convey urgency without inducing anxiety when action may be indicated and consider respect and empathy for the patients’ symptoms when action may not be indicated to ensure that they do not feel unheard and are empowered to report new or worsening symptoms.

## Appendix

Multimedia Appendix 1 Semistructured interview guide questions for patients and health professionals.

## Abbreviations

CDC: Centers for Disease Control and Prevention
EPDS: Edinburgh Postnatal Depression Scale
mHealth: mobile health
PRD: pregnancy-related death
PRO: patient-reported outcome
REDCap: Research Electronic Data Capture
UWS: Urgent Maternal Warning Signs

## Data Availability

The data sets generated and analyzed during this study are not publicly available due to institutional review board regulations but are available from the corresponding author on reasonable request.
