# Supplementary material for: Understanding Symptom Self-Monitoring Needs Among Postpartum Black Patients: Qualitative Interview Study
Source: J Med Internet Res. 2024 Apr 26;26:e47484. doi: 10.2196/47484 (PMC11087860; doi:10.2196/47484)
Supplement: Multimedia Appendix 1 [file jmir_v26i1e47484_app1.docx]

**Multimedia Appendix 1**. Semistructured interview guide questions for patients and health professionals.

*These interviews served as general guides, not verbatim scripts

Postpartum experience

- *Just so we understand your experience, was your most recent pregnancy your first child?*
- *What kind of health professionals did you work with before or after the birth of your baby?*
  - Examples: Doctors, midwives, nurse practitioners, physician assistants, nurses, doulas, lactation consultants
- *Thinking back to when you were leaving the hospital, what were YOU most worried about [related to your own health]?*
- *Did health professionals tell you things to watch out for related to your health?*
  - *Was the information: written? Verbal? A video?*
  - *Who did this information come from?* [For example, did you have the chance to talk with a social worker].
  - *Was this information easy to understand? Why or why not?*
  - *Are there ways they could have made this information easier to understand or use?*
  - *Did you have the opportunity to ask questions and discuss your concerns?*
  - *Do you remember if you ever looked at this information after you got home from the hospital? Why or why not?*
- *If you can think back to your first pediatrician or OB visit, how did you feel mentally and physically around that time?*
- [Probes] *For example, did you have any of the following?*
  - Headache, changes in your vision, dizziness, or fainting; extreme swelling or redness in your hands, feet, or face; Severe nausea and throwing up?
  - Chest pain or fast beating heart, trouble breathing, dizziness, leg pain or swelling
  - Severe nausea and throwing up, having vaginal bleeding or discharge
  - Having trouble sleeping when the baby sleeps, having trouble “turning off your mind”, feeling overly tearful or irritable, having trouble enjoying things you normally enjoy
- *Did you ever feel like you should get help [from a medical professional] for the way you were feeling?*
  - *What were your options for getting help?*
- *What did you ultimately decide to do?*
- *What information led you to that decision* (e.g. things your doctor had told you, things you know about your health)?
  - Were there past experiences that affected your choice?
  - Did you ask other people (i.e. friends or family)?
  - Were there other factors (e.g. insurance, transportation support) that made your decision easier or harder?
- *Were you able to discuss how you were feeling after having your baby with your doctor, your child’s doctor, or another health professional (e.g., lactation consultant, doulas)?*
- *What was their response?*
  - Was that helpful for the problem you were experiencing?
- *If you think about any symptoms, you might have had after you had your baby or even during pregnancy, do you think there could have been better ways to explain to you what issues the providers were looking for or when you should call your doctor?*
- *Is there anything else about your experience after giving birth that you think would allow us to better help women deal with any health problems they have during that time?*

Information use preferences:

Now, I am going to ask a few questions about where you get information about your and your child’s health:

- What kinds topics have you read about related to pregnancy, your own health, or caring for your child?
- Did any of this information require that you provide data about your own or your child’s health?
- How did you get this information (phone, internet, etc.)?
- Where did this information come from (social media, your doctor, friends/family)?
- How did you decide or know if the information was trustworthy?

Design needs specific questions

- One thing we are thinking about doing for this project is giving moms an app or sending text messages to ask about any health problems they may have and provide information about these symptoms through the app or messages. For example, when to call their doctor and what to say. We do not know exactly how this would look, and we want to learn from you about what you think is or isn’t helpful. Is the text messaging or app something you think you would have used? Why or why not?

| **Design need/ Feature** | **Question** |
| --- | --- |
| General | *What would make you want to use a system like this…*   - *At the outset* (based on how it is introduced to you or marketed)? - *The features it contains* - *Other things* [follow up with items below as needed] |
| Symptom reporting | - *How would you feel about providing this kind of information through your phone? Would you be comfortable doing it?* - *How often would you be willing to report your symptoms (once per day, as needed when you aren’t feeling well)?* - *When (what time of day or during what kind of activity) would you like to report symptom information?* - *How would you want to report them (survey, choose from a list, etc.)?* - *Would reminders to open the system or report symptoms be helpful?* - *Would it be helpful to customize when these reminder would happen?* |
| Symptom response messages | *Based on the symptoms you report, we are guessing there are three kinds of messages options: 1) no action needed, 2) recommendation to call your OB’s office, or, hopefully rarely, 3) recommendation to go to or call the hospital.*   - No action needed   - *What would be helpful or re-assuring for you to hear if there is no action recommended based on the symptom you report?*   - *Would you like to also get advice about things you can do on your own to cope with these symptoms?* - Call your OB’s office   - *What information would help encourage you to call your OB’s office?*   - *How would you like the call to happen (e.g. schedule a call, have them call you, link to a chat)?* - Call/go to the hospital   - *What information would help encourage you to call or go to the hospital?*   - *What kinds of worries would you have about going to the hospital?* |
| Provider involvement | - *How involved would you like your OB to be? Do you know they’ve received information about the symptoms you’ve reported?* - *Is it helpful to you to have this application linked to your patient portal?* |
| Additional resources | *What other kinds of resources would be helpful to include in the system?* [Examples]   - Resources to help with how you are feeling mentally - Lactation resources - Information about your baby - Information about your doctor’s office - Information about available emergency care near you - Other social services (food, support groups) - Summaries of your symptom reports to review in visits with a nurse or provider   *For these resources, is it important that they are:*   - *Tailored to where you live* - *Tailored to your insurance* |
| Friend/family support | *We have also discussed that some people may find it helpful to also have trusted friend or family members have access to educational information (but not report symptoms).*   - *What do you think about this?* - *How would you (if at all) like to involve a trusted friend or family member (for example, a partner, a parent, or a friend)?* |

**Health professionals (obstetric and mental health) semi-structured interview guide**

Post-partum Symptom Monitoring Patient self-monitoring

1. What kinds of concerning symptoms have you had mothers report issues within the postpartum period?
   - Probe: This can include both physical and mental health symptoms.
2. Are there specific cases that stick out where the issues would have been more easily resolved had the patient sought care sooner? Please provide examples if you can.
3. What are the most common symptoms that are frequently overlooked by mothers in the postpartum period?
   - From your clinical experience, what would you say are the most common barriers that mothers have with identifying and responding to these symptoms?
   - Probe: Do you think they know what symptoms to look for?
   - Do you think they aren’t sure what’s normal and what’s not?
4. What are the barriers that women face in seeking care in response to their symptoms?
   - Have you witnessed any experiences where there was incongruence the patient report of the symptom vs. the provider perception of the symptom?
5. Do you have any strategies you use personally or have seen in clinical practice to help patients know how to self-monitor their symptoms in the postpartum period?
   - Probe: Do you do this/did you see this strategy used with all patients or only certain patients?

If not addressed, for 4 and 5, specifically probe about:

- - Discrimination probes:
    - Do you think discrimination (perceived or past) plays a role in self-monitoring/care seeking? How so?
  - Previous experience with healthcare system probes:
    - Have some patients had positive experiences with the healthcare system that have allowed them to better self-monitor?
    - Have some patients had negative experiences with the healthcare system that have hindered their self-monitoring/care seeking behaviors?
  - Self-perception probes:
    - Have you noticed issues such as not wanting to say they need help, be perceived as weak, over-confidence?
  - Socio-cultural probes:
    - Have you noticed barriers/facilitators related to things friends/family members tell the patient?
    - Have you noticed barriers/facilitators related to role of their partners or family members play (overly active, not active enough)
    - Have you noted patients being concerned with friends/family members will perceive them?
    - Have you encountered patients with the perception that medical issues may be handled by non-medical remedies (e.g., folk remedies)?
  - Informational issue probes:
    - Do patients seem to have issues with the information they are given (e.g., at hospital discharge)? Is it difficult for them to understand?
    - Do you think patients know what symptoms to be looking for?
    - Do you think patients know what their options are for seeking help?
  - Structural/organizational issues:
    - Do you think financial/insurance related concerns affect care seeking behaviors?
    - What about issues with transportation?
    - Or competing demands such as caring for other children, work, etc.?
    - Do you think patients know who to call? Or what to do if they cannot get through? Using the portal vs not?

Clinical evaluation of patient symptoms

1. What symptoms cause you to worry with women in the postpartum period? [Allow for initial answer and then CDC urgent maternal warning signs handout]
2. Are there symptoms that are commonly reported but not as problematic as the patient perceives?
3. How could patients better describe their symptoms to improve your clinical decision-making?
